# Update in Laboratory Diagnosis of Thalassemia

**DOI:** 10.3389/fmolb.2020.00074

**Published:** 2020-05-27

**Authors:** Thongperm Munkongdee, Ping Chen, Pranee Winichagoon, Suthat Fucharoen, Kittiphong Paiboonsukwong

**Affiliations:** ^1^Thalassemia Research Center, Institute of Molecular Biosciences, Mahidol University, Nakhon Pathom, Thailand; ^2^Guangxi Key Laboratory of Thalassemia Research, Guangxi Medical University, Nanning, China

**Keywords:** thalassemia, hemoglobinopathies, diagnosis, hemoglobin analysis, DNA analysis

## Abstract

Alpha- and β-thalassemias and abnormal hemoglobin (Hb) are common in tropical countries. These abnormal globin genes in different combinations lead to many thalassemic diseases including three severe thalassemia diseases, i.e., homozygous β-thalassemia, β-thalassemia/Hb E, and Hb Bart’s hydrops fetalis. Laboratory diagnosis of thalassemia requires a number of tests including red blood cell indices and Hb and DNA analyses. Thalassemic red blood cell analysis with an automated hematology analyzer is a primary screening for thalassemia since microcytosis and decreased Hb content of red blood cells are hallmarks of all thalassemic red blood cells. However, these two red blood cell indices cannot discriminate between thalassemia trait and iron deficiency or between α- and β-thalassemic conditions. Today, Hb analysis may be carried out by either automatic high-performance liquid chromatography (HPLC) or capillary zone electrophoresis (CE) system. These two systems give both qualitative and quantitative analysis of Hb components and help to do thalassemia prenatal and postnatal diagnoses within a short period. Both systems have a good correlation, but the interpretation under the CE system should be done with caution because Hb A2 is clearly separated from Hb E. In case of α-thalassemia gene interaction, it can affect the amount of Hb A2/E. Thalassemia genotypes can be characterized by the intensities between alpha-/beta-globin chains or alpha-/beta-mRNA ratios. However, those are presumptive diagnoses. Only DNA analysis can be made for specific thalassemia mutation diagnosis. Various molecular techniques have been used for point mutation detection in β-thalassemia and large-deletion detection in α-thalassemia. All of these techniques have some advantages and disadvantages. Recently, screening for both α- and β-thalassemia genes by next-generation sequencing (NGS) has been introduced. This technique gives an accurate diagnosis of thalassemia that may be misdiagnosed by other conventional techniques. The major limitation for using NGS in the screening of thalassemia is its cost which is still expensive. All service labs highly recommend to select the technique(s) they are most familiar and most economic one for their routine use.

## Introduction

Hemoglobinopathies may be roughly divided into two groups, the structural hemoglobin (Hb) variants (abnormal Hb) and the thalassemias. These structural Hb variants are commonly caused by single amino acid substitutions in the α or β globin chains. Most of these abnormal Hb do not have clinical symptoms; however, some of these mutations may change the functional properties or stability of the Hb and lead to a clinical disorder. The thalassemias result from defective globin chain production. They are classified into the α, β, δβ, and δβγ-thalassemias according to the particular globin chains that are defectively synthesized. From a public health point of view, only the α and β-thalassemias are common to be of importance (Calzolari et al., 1999; Weatherall and Clegg, 2001).

The major pathophysiological change of thalassemias is imbalanced globin-chain production. This leads to the destruction of the red blood cell precursors in the bone marrow or peripheral blood result in chronic anemia, splenomegaly, and skeletal deformity due to expansion of the bone marrow (Weatherall, 1998). The homozygous or compound heterozygous states for β-thalassemia have a variable course; however, death occurs mostly in the first few years of life without transfusion. With adequate transfusion and iron chelator administration, the thalassemia patients may have good development and can survive into adulthood. Beta-thalassemias of intermediate severity, such as β-thalassemia/Hb E, have a wide range of clinical spectrum from a condition that is compatible with normal survival and growth into adult life without treatment to a transfusion-dependent thalassemia (TDT). The reasons for this clinical heterogeneity are not fully understood (Olivieri, 1999; Barbara, 2006).

The α-thalassemias are equally heterogeneous. The milder forms (termed α-thalassemia 2 or α^+^-thalassemias) result from one α globin gene deletion, produce a mild anemia in their homozygous states. While α-thalassemia 1 or α^0^-thalassemia is associated with an absence of α globin chain synthesis because of the deletion of the two α globin genes on the same chromosome. In homozygous states, it results in the most severe form of thalassemia, namely, Hb Bart’s hydrops fetalis. The compound heterozygous states for α thalassemia 2 and α-thalassemia 1 result in Hb H disease which varies in severity; at the more severe end, it may be a TDT.

The thalassemias are extremely heterogeneous at the molecular level; over 200 different mutations of the β globin genes have been found in patients with β-thalassemia, and the α-thalassemias are almost as varied in their molecular pathology. However, global population seems to carry a few common mutations that are unique to a particular area, together with varying numbers of rare ones.

Here we will describe conventional methods for thalassemia diagnosis which first characterized subjects with phenotypic traits associated with thalassemia by using hematological (red blood cell indices) and biochemical tests (Hb analysis) and subsequent DNA analysis for definitive diagnoses. However, this diagnosis approach would not detect individuals with normal or borderline red blood cell indices and/or HbA2 levels which are “silent” forms of thalassemia (Yilmaz et al., 2019). In addition, at least 1,800 mutations causing thalassemia or abnormal hemoglobin variants have been characterized to date, the identification of mutation in samples from subjects suspected of having hemoglobinopathies may require labor-intensive methods (Singh et al., 2020). The application of new technology and high-throughput molecular approaches such as next-generation sequencing (NGS) for screening and accurate diagnosis of hemoglobinopathies is feasible (Li et al., 2020; Ruengdit et al., 2020; Wang et al., 2020; Zhao et al., 2020).

## Laboratory Diagnosis of Thalassemia

Thalassemias and abnormal hemoglobin diagnoses require a combination of laboratory tests including the measurement of red blood cell indices by automatic hematology analyzer, Hb analysis, and quantification of Hb A2 and Hb F. The high-performance liquid chromatography (HPLC) and capillary zone electrophoresis (CE) system can distinguish thalassemic diseases and the carriers. It has been widely used to replace the manual technique. These systems give both qualitative and quantitative analyses of Hb components with good precision and reproducibility. They have enabled us to do both prenatal and postnatal diagnoses of thalassemia within the few minutes. Specific thalassemia mutation can be detected by DNA analysis, and various techniques have been used for point mutation detection. Moreover, thalassemia genotyping can be carried out by real-time polymerase chain reaction (PCR) follows by melting curve analysis. Cases that mutation cannot identify by previous molecular analysis technique will be sent for DNA sequencing. And in the last few years, the genome sequencing by NGS has been applied for thalassemia diagnosis as well.

## Automatic Hemoglobin Analyzers

Because of less globin production, the thalassemic red blood cells showed microcytic and hypochromic. However, Hb, mean corpuscular volume (MCV), and mean corpuscular Hb (MCH) cannot discriminate between thalassemia trait and iron deficiency or between α- and β-thalassemia (England and Fraser, 1973). Hb analysis is needed to determine α- and β-thalassemia carriers and disease. Automatic HPLC and CE system are sensitive and precise methods for qualitative and quantitative analyses of Hb components in red blood cells (Stephens et al., 2015). The HPLC system is cation exchange and use two dual piston pumps to set gradient sodium phosphate buffers of increasing ionic strength to pass through a column spherical cation exchange resin during a 6.5 min. Hemolysate samples are determined by spectrophotometer that read double wavelength at 415 and 690 nm. The resulting chromatograms are separated in retention time (RT). Similarity, the CE system is based on capillary electrophoresis in free solution from cathode to anode. Hb components are separated in silica capillaries by their electroosmotic flow and at a high voltage (9,800 V) in electrophoretic mobility in an alkaline buffer. The photometry at an absorbance wavelength 415 nm was used to directly detect Hb fractions. The resulting electrophorograms are divided into 15 zones. Several publications on the automatic hemoglobin analyzers have shown their effectiveness in the investigation of thalassemia and hemoglobinopathies for prenatal and postnatal diagnoses (Tan et al., 1993; Borbely et al., 2013; Khongthai et al., 2019; Li et al., 2019).

## Hemoglobin Analysis in Adult

**FIGURE 1:**
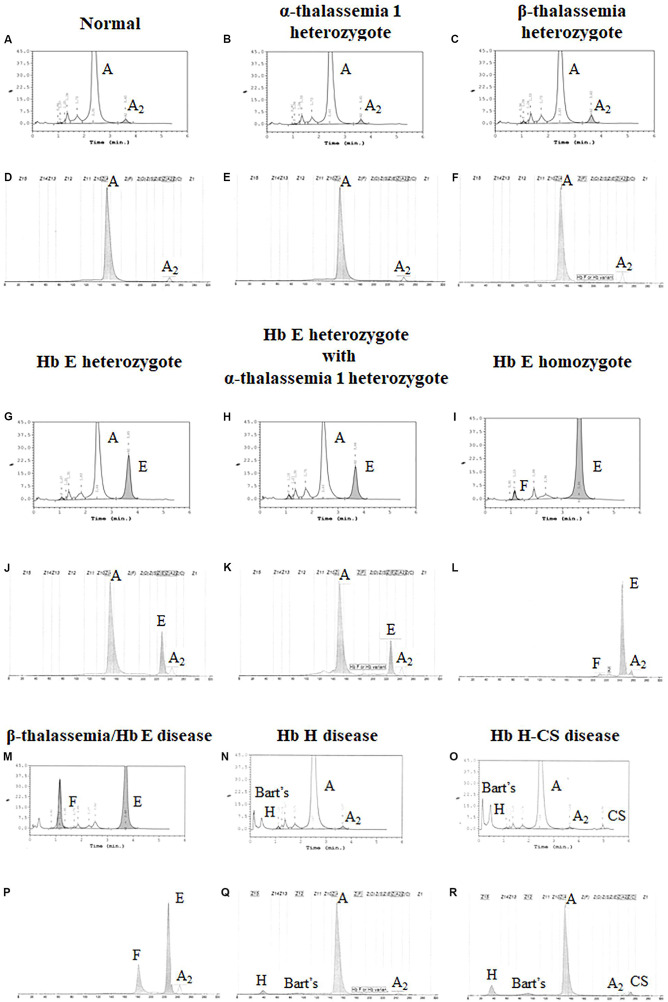
Pattern of hemoglobin analysis in adult blood by the high-performance liquid chromatography (HPLC) system **(A–C,G–I,M–O)** compared with the capillary zone electrophoresis (CE) system **(D–F,J–L,P–R)**.

Both systems give a good correlation for thalassemia diagnosis in adult. Normal blood samples had Hb concentration = 12 g/dl, MCV = 80 fL, MCH = 27 pg, and HbA2 = 3.5% (Figures 1A,D). Thalassemia carriers presented normal Hb concentration level but show low MCV and MCH. Alpha-thalassemia carriers had Hb A2 = 3.5% (Figures 1B,E), but β-thalassemia carrier had Hb A2 > 3.5% (Figures 1C,F). Under the HPLC system, Hb A2 and Hb E co-elute at the same RT, but in the CE system, Hb A2 and Hb E are clearly separated, zone three for Hb A2 and zone four for Hb E. The average amount of Hb E was 27.8 ± 7.5% by HPLC and 25.6 ± 1.4% by CE. In addition, Hb A2 is detected with the average amount of Hb A2 3.5 ± 0.4% under the CE system. In Hb E homozygote, Hb A2 + E was 90.2 ± 4.9% by HPLC and Hb A2 was 4.1 ± 0.8% and Hb E was 92.9 ± 3.3% by CE (Figures 1I,L). In β-thalassemia/Hb E disease, Hb A2 + E was 59.4 ± 12.9% by HPLC and Hb A2 was 4.9 ± 1.6% and Hb E was 50.3 ± 13.8% by CE (Figures 1M,P). In fact, the amount of Hb A2/E is more confusing in the double heterozygote of Hb E and α-thalassemia cases with different numbers of defective α-globin gene. For example, the double heterozygote with Hb E and α-thalassemia 1 presented low Hb E levels, 21.9 ± 0.6% and 16.3 ± 0.8% by HPLC and CE, respectively (Figures 1G,H,J,K). The CE system demonstrated Hb E level less than HPLC because of the separated zones of Hb A2 and E. Therefore, criteria for Hb E diagnosis must combine % Hb A2 and E to reduce misdiagnosis (Table 1).

**TABLE 1:** Hemoglobin analysis of adult blood.

Phenotype	Number	Hb Type	Hb A_2_ %		Hb E %		Hb F %	
			HPLC	CE	HPLC	CE	HPLC	CE
Normal	45	A_2_A	2.6 ± 0.4	2.5 ± 0.4	–	–	0.5 ± 0.7	0.1 ± 0.2
α-thalassemia 1 heterozygote	36	A_2_A	2.3 ± 0.2	2.3 ± 0.2	–	–	0.5 ± 0.7	0.3 ± 0.5
β-thalassemia heterozygote	69	A_2_A	5.5 ± 1.3	5.4 ± 0.5	–	–	1.5 ± 1.4	0.9 ± 1.4
Hb E heterozygote	85	EA	Not detected	3.5 ± 0.4	27.8 ± 7.5	25.6 ± 1.4	1.2 ± 0.9	0.4 ± 0.8
Hb E heterozygote with α-thalassemia 1 heterozygote	6	EA	Not detected	4.0 ± 0.3	21.9 ± 0.6	16.3 ± 0.8	0.9 ± 0.6	0.5 ± 0.8
Hb E homozygote	56	EE	Not detected	4.1 ± 0.8	90.2 ± 4.9	92.9 ± 3.3	4.3 ± 2.7	2.5 ± 3.1
β-thalassemia/Hb E disease	48	EF	Not detected	4.9 ± 1.6	59.4 ± 12.9	50.3 ± 13.8	31.1 ± 14.5	36.8 ± 13.3
Hb H disease	26	A_2_A Bart’s H	1.6 ± 1.2	1.0 ± 0.2	–	–	0.6 ± 0.6	0.2 ± 0.3
Hb H-CS disease	9	CSA_2_A Bart’s H	ND	0.7 ± 0.5	–	–	ND	1.0 ± 0.6
Hb CS homozygote	10	CSA_2_A	ND	1.3 ± 0.6	–	–	ND	0.8 ± 0.8

**Phenotype**	**Number**	**Hb Type**	**Hb Bart’s %**		**Hb H %**		**Hb CS %**	
			**HPLC**	**CE/Undetected number**	**HP**	**CE/undetected number**	**HPL**	**CE/undetected number**

Normal	45	A_2_A						
α-thalassemia 1 heterozygote	36	A_2_A						
β-thalassemia heterozygote	69	A_2_A						
Hb E heterozygote	85	EA						
Hb E heterozygote with α-thalassemia 1 heterozygote	6	EA						
Hb E homozygote	56	EE						
β-thalassemia/Hb E disease	48	EF						
Hb H disease	26	A_2_A Bart’s H	Found	1.1 ± 0.7/14	Found	6.7 ± 4.8/0		
Hb H-CS disease	9	CSA_2_A Bart’s H	Found	4.2 ± 4.1/3	Found	11.3 ± 6.5/3	Found	2.6 ± 1.4/1
Hb CS homozygote	10	CSA_2_A	Found/Not found		Found/Not found		Found	3.5 ± 2.5/0

*ND, not determined.*

In addition, the two systems can also detect Hb Bart’s, Hb H, and Hb CS in Hb H and Hb H-CS diseases of which the hemoglobin peaks were shown in Figures 1N,O,Q,R. The HPLC system demonstrates the qualitative Hb H and Hb Bart’s peaks and quantitative Hb CS (Figures 1N,O). However, CE system measures quantitatively Hb H, Hb Bart’s, and Hb CS (Figures 1Q,R and Table 1). Nevertheless, Hb Bart’s, Hb H, and Hb CS are unstable and a decreasing level may be found in the blood sample that was kept for a long storage especially at high temperature (Fucharoen et al., 1998b; Winichagoon et al., 2008; Munkongdee et al., 2011; Kingchaiyaphum et al., 2020).

## Hemoglobin Analysis in Newborn

**FIGURE 2:**
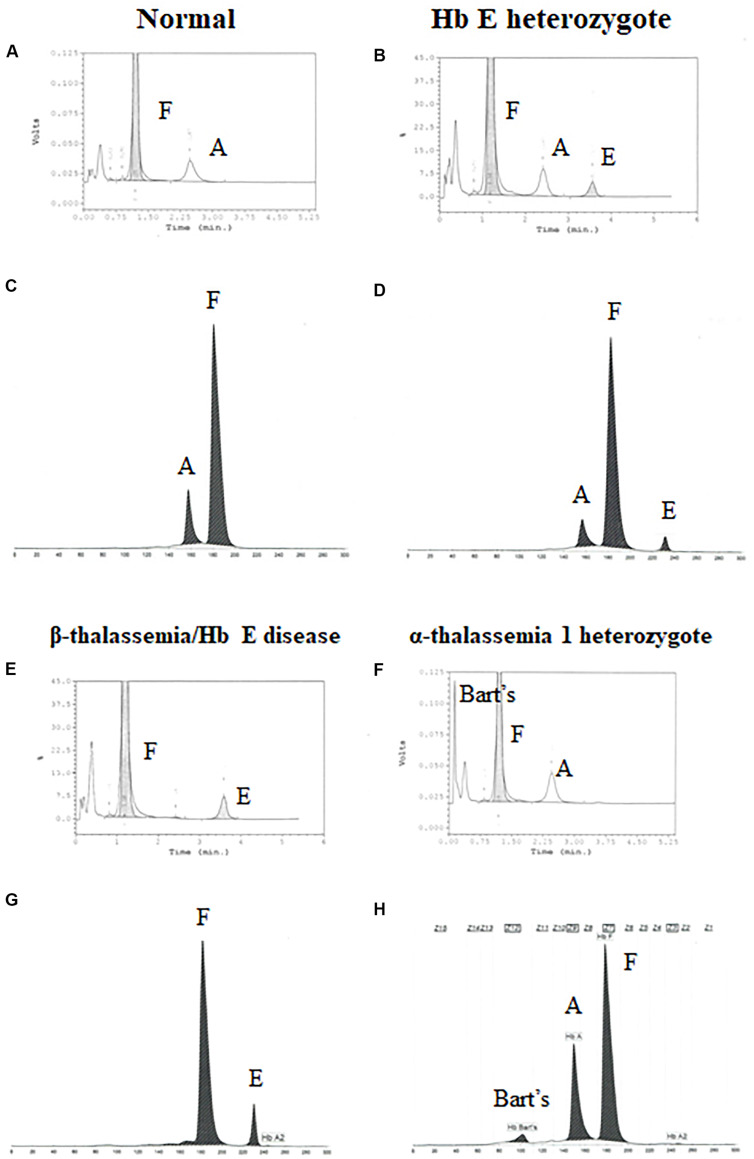
Pattern of hemoglobin analysis in cord blood by the high-performance liquid chromatography (HPLC) system **(A,B,E–F)** compared with the capillary zone electrophoresis (CE) system **(C,D,G–H)**.

Newborns screening for thalassemia can also be diagnosed by HPLC and CE systems. Normal newborn had normal Hb concentration 15.5 ± 1.3 g/dl, MCV 110.0 ± 4.8 fL, and MCH 35.9 ± 1.4 pg and Hb analysis presented FA (Figures 2A,C and Table 2). Newborns with β-thalassemia heterozygote also has the normal hematological parameters because the β-globin gene is not fully expressed at birth (Figures 2B,D,E,G and Table 2) (Fucharoen et al., 1998b; Winichagoon et al., 2008; Munkongdee et al., 2011).

**TABLE 2:** Hemoglobin analysis of cord blood.

Phenotype	Number	Hb Type	Hb (g/dL)	MCV (fL)	MCH (pg)	Hb A %		Hb F %		Hb E %		Hb Bart’s %	
						HPLC	CE	HPLC	CE	HPLC	CE	HPLC	CE
Normal	339	FA	15.5 ± 1.3	110.0 ± 4.8	35.9 ± 1.4		18.3 ± 6.8		81.6 ± 6.9			0.7 ± 0.4	0.0 ± 0.1
Hb E heterozygote	86	EFA	15.4 ± 1.4	108.6 ± 5.0	35.4 ± 1.6		10.0 ± 3.8		86.1 ± 4.8		3.8 ± 1.3	0.7 ± 0.4	0.0 ± 0.1
Hb E heterozygote with α-thalassemia 1 heterozygote t	6	EFA Bart’s	14.4 ± 0.4	87.9 ± 3.6	28.0 ± 1.4		7.6 ± 4.0		83.1 ± 2.7		4.4 ± 4.3	8.6 ± 1.7	4.8 ± 1.0
Hb E homozygote	9	EF	14.5 ± 2.1	103.0 ± 6.7	34.0 ± 2.5		ND	81.4 ± 4.5	ND	8.0 ± 3.5	ND		ND
β-thalassemia/Hb E disease	4	EF	14.8 ± 2.3	105.7 ± 6.3	34.1 ± 2.1		0	93.0	90.2 ± 4.5	2.0	9.2 ± 4.0	1.0	0 ± 0
α-thalassemia 1 heterozygote	17	FA Bart’s	13.4 ± 1.0	89.8 ± 3.4	28.0 ± 1.2		23.6 ± 5.3		71.5 ± 5.4			9.2 ± 1.1	4.6 ± 0.5
Hb H disease	2	FA Bart’s	74	22	30	43.5	ND	52.0	ND	0	ND	24.2	ND
Hb H-CS disease	3	CSFA Bart’s	12.0 ± 0.1	97.2 ± 1.7	26.4 ± 0.5	ND	20.0 ± 8.2	ND	46.3 ± 7.0		0	ND	32.1 ± 3.1
Hb CS homozygote	1	CSFA Bart’s	12.3	115.0	30.0	13.9	ND	78.5	ND	0	ND	15.0	ND

*ND, not determined.*

In contrast, newborns with α-thalassemia have the abnormal hematological parameters, similarity with adults, because the α-globin gene is expressed in the fetus. Specifically, the MCVs and MCHs of α-thalassemia newborns are significantly lower than those of the normal newborn. In addition, Hb Bart’s was obscurely presented in newborn associated with α-thalassemia (Figures 2F,H and Table 2) (Fucharoen et al., 1998b; Winichagoon et al., 2008; Munkongdee et al., 2011).

## Interactions Between α-Thalassemia and β-Thalassemia

Up to now, more than 1,800 mutations causing hemoglobinopathies have been discovered (Huisman et al., 1997; HbVar, 2019). These mutated α and β globin genes in different combinations lead to over 60 thalassemic diseases (Fucharoen and Winichagoon, 1997; Fucharoen et al., 1998a). The degree of disease severity depends on the complexity of gene interaction, and even identical genotypes patients can have remarkably different levels of severity. This can lead to the difficulty on identification of high-risk pregnancies and provide appropriate genetic counseling for both treatment and prenatal diagnosis.

The variability in severity of thalassemias, especially β-thalassemia, involves many gene loci, some of which are directly involved with defects in α, β, or γ globin synthesis, whereas others, related to other genes such as *KLF1*, *BCL11A* (Sripichai and Fucharoen, 2016). A good example is observed in β-thalassemia/Hb E patients who co-inherit α-thalassemia as they will have less unmatched α-globin chains, which result in less symptoms (Kanavakis et al., 1982; Wainscoat et al., 1983; Winichagoon et al., 1985). On the other hand, the co-inheritance of triplicated α-globin genes (ααα) may lead to an increase of globin chain imbalance and severe anemia in β-thalassemia (Galanello et al., 1983).

**FIGURE 3:**
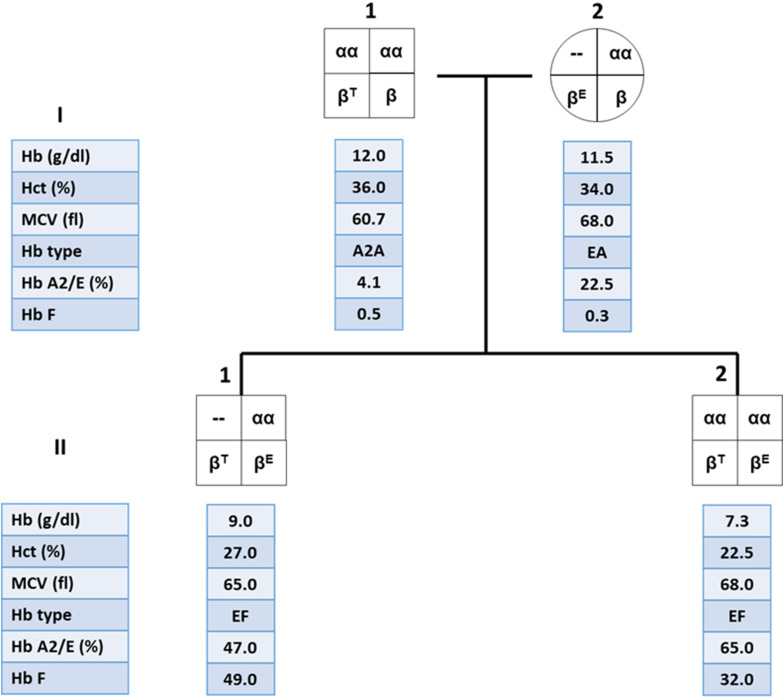
Family pedigree showing the co-inheritance of α-thalassemia 1 in β-thalassemia/Hb E patient (II.1) with high hemoglobin (Hb) level compared to his younger brother who is a β-thalassemia/Hb E patient without α-thalassemia (II.2).

These findings are important in genetic counseling especially in the high risk couples for β-thalassemia/Hb E who are performing prenatal diagnosis. An example of the family pedigree, including hematological data of the high-risk couples and their offspring, is shown in Figure 3. The mother is double heterozygous for Hb E and α-thalassemia 1, while the father is β-thalassemia heterozygote. It is important to characterize the father’s specific β-thalassemia mutation. If the mutation is a β^0^-thalassemia, there is a 1/4 chance that the future child would be a compound heterozygote for β^0^-thalassemia/Hb E. This would result in a moderate or severe clinical symptoms, with iron overload and possible TDT. B eta^0^-thalassemia/Hb E child who co-inherit with α-thalassemia 1 would be expected to have less symptoms than β^0^-thalassemia/Hb E child who does not carry α-thalassemia and may result in a TDT. Thus, the recommendation for both parents would be DNA analysis for α globin with additional β-globin DNA analysis for the father. Prenatal diagnosis would allow high-risk couples to determine possible adverse outcomes in their pregnancies.

Hb analysis generally do not contribute to the diagnosis of mild forms α-thalassemia, in which one or two (cis or trans) α-globin genes are deleted. The excess β-globin chains (Hb H molecule) in these α-thalassemia cannot be visualized by Hb analysis. Thus, α-thalassemia is often diagnosed by exclusion, when a subject with microcytic red blood cells, normal Hb analysis and normal iron studies is presumed to have α-thalassemia. Alpha-thalassemia may also be masked in the presence of β-thalassemia which also have microcytic red blood cells. For these subjects, family study and DNA analysis may be the definitive diagnosis, and this is important for genetic counseling.

## Molecular Analysis for α and β-Thalassemia Mutations

The advent of the PCR has enabled screening for single-base mutations to become simpler (Eisenstein, 1990). Most of thalassemia mutations are point mutation. Point mutations are considered to be single-base substitutions or minor insertions or deletions. Table 3 showed a summary of common DNA techniques used for point mutation detection. In this paper, we will briefly discuss only common DNA techniques, namely, allele-specific PCR, reverse dot blot (RDB) analysis, real-time PCR with melting curve analysis, and DNA sequencing.

**TABLE 3:** Summary of common molecular technique used for point mutation detection.

Known mutation	Unknown mutation
Gel electrophoresis	Mismatched analysis
Allele-specific PCR	Denaturing gradient gel electrophoresis
Dot blot analysis	DNA sequencing
Real-time PCR with melting curve analysis	

*All of these techniques developed after gene amplification by polymerase chain reaction (PCR).*

## Allele-Specific PCR

**FIGURE 4:**
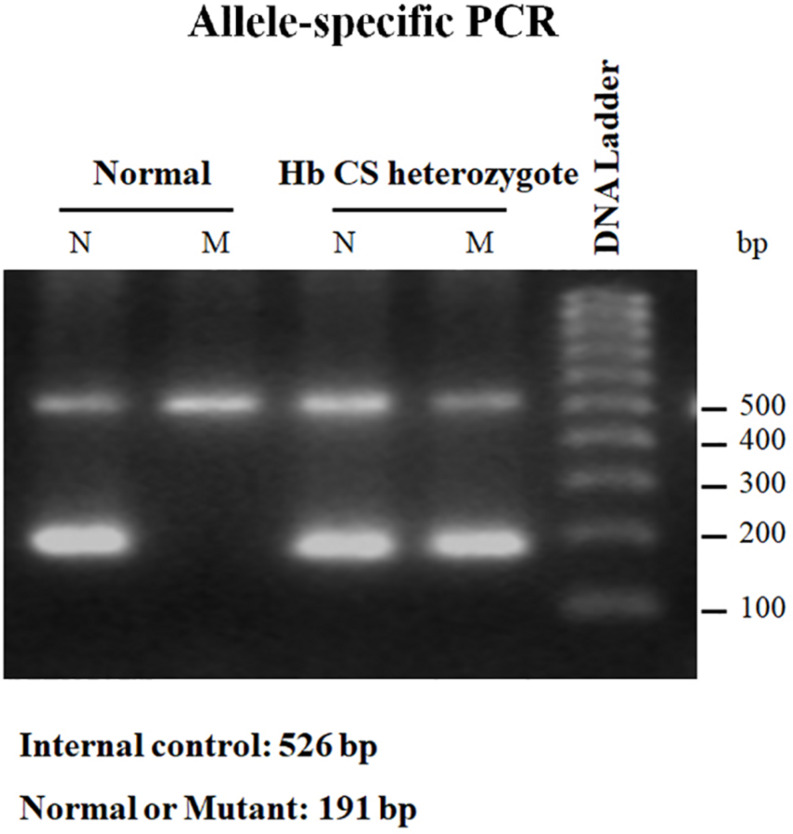
Result of allele-specific PCR showing PCR product of normal individual which can be seen only in the reaction employing the wild-type (N) primer set. While Hb CS heterozygote will generate a band using both wild-type (N) and mutant (M) primer set. Internal control was exhibited at 526 bp.

This technique employs two primers identical in sequence except for the 3′-terminus base, one of which is complementary to the wild-type and the other for the mutant base; a common primer for the opposite strand must of course be used as well. For primer extension to occur using *Taq* polymerase which has no 3′–5′ exonuclease (proofreading) activity, perfect matching of the primer 3′-terminus with the DNA template must occur. With a normal individual, PCR product will be seen only in the reaction employing the wild-type primer set. A heterozygote will generate a band using both wild-type and mutant primer set, and an individual with homozygous mutation will be negative with the normal and positive with the mutant primer set (Figure 4) (Suwannakhon et al., 2019).

## Reverse Dot-Blot Analysis

The suspected mutation can be identified by hybridization of an allele-specific oligomer (ASO) DNA probe with the PCR product which is immobilized on a membrane filter sheet as a dot. The ASO probe can be radiolabeled with ^32^P for autoradiography or has attached reporter groups (biotin, digoxigenin, or an enzyme such as horseradish peroxidase) which can subsequently be visualized in a chemiluminescent or colorimetric reaction. Commercial kits have made these non-radioactive detection methods quite a routine procedure. For each mutation, two hybridization reactions need to be conducted, one with the probe for the mutant sequence and the other with the probe for the normal sequence. The stringency of hybridization has to be optimized for each ASO probe.

**FIGURE 5:**
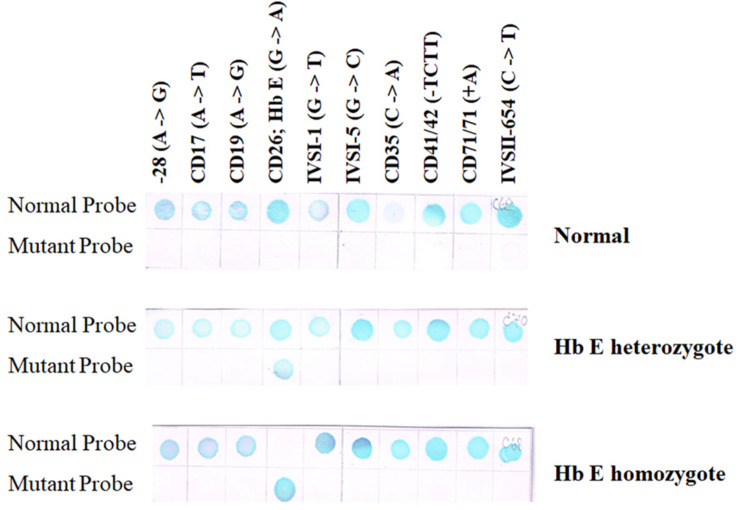
Result of RDB showing a result of normal individual will give positive results with each wild-type sequence but not with any mutant probe (upper panel). Heterozygotes show a reaction with a single mutation dot in addition to the normal dots (middle panel), whereas homozygotes will give a positive dot with that mutant probe but not with its corresponding normal sequence (two mutant dots will be seen if the individual carries two different mutations) together with positive spots for the remaining normal probes (lower panel).

A reverse dot-blot analysis has been developed (Maggio et al., 1993; Winichagoon et al., 1995). The ASO probes contain an amino group at the 5′-terminal base which enables them to be covalently attached onto a nylon membrane strip. This is then hybridized with amplified DNA which has been labeled with biotin for colorimetric detection. A normal individual will give positive dots with each wild-type probe but not with any mutant probe (Figure 5 upper panel). Heterozygotes exhibit a positive with one mutation dot in addition to the normal dots (Figure 5 middle panel), whereas homozygotes for mutation will give a positive dot with that mutant probe but not with its corresponding normal sequence together with positive spots for the remaining normal probes (Figure 5 lower panel). A critical requirement for this technique is the optimization of washing temperature for all of the probes. This can be achieved by optimizing the length of each ASO probe.

## Real-Time PCR With Melting Curve Analysis

The conventional PCRs give a clear result, but it requires labor-intensive and time-consuming in post-PCR processing steps. The real-time PCR or quantitative PCR (qPCR) is widely used to detect, characterize, and quantify nucleic acids. It is high throughput, automation, and low risk of post-PCR contamination. Currently, the application of real-time PCR with melting curve analysis for thalassemia diagnosis is based on two general approaches, intercalating dye assays and probe-based assays, obtaining a fluorescent signal from the synthesis of product in PCR.

**FIGURE 6:**
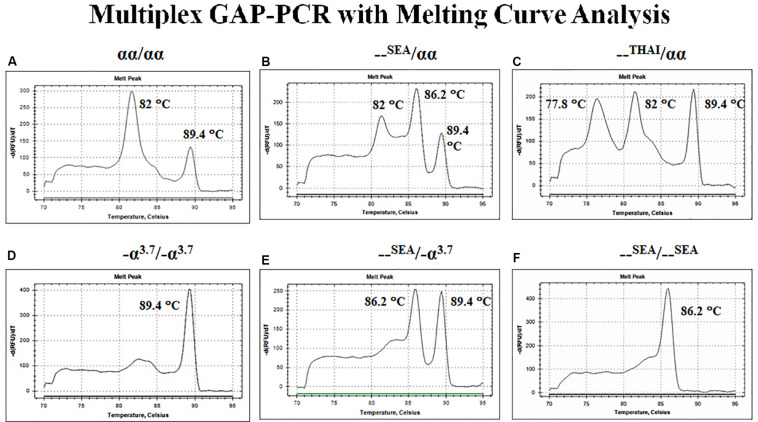
Multiplex GAP–PCR with melting curve analysis for α-thalassemia genotyping. Panel **(A)** shows two peaks of α2-globin and ψζ-globin fragments from normal globin genotype. Panel **(B)** shows three peaks of α2-globin, –^SEA^, and ψζ-globin from α-thalassemia 1 heterozygote (–^SEA^ type). Panel **(C)** shows three peaks of –^THAI^, α2-globin, and ψζ-globin from α-thalassemia 1 heterozygote (–^THAI^ type). Panel **(D)** shows a single peak of -α^3^.^7^ from α-thalassemia 2 homozygote. Panel **(E)** shows two peaks of –^SEA^ and ψζ-globin from deletional Hb H disease. Panel **(F)** shows a single peak of –^SEA^ from Hb Bart’s hydrops fetalis (α-thalassemia 1 homozygote; –^SEA^ type).

The first approach depends on the fluorescent DNA-intercalating dyes such as SYBR Green I to bind with double-stranded DNA (dsDNA) and undergo a conformational change that leads to an increase in their fluorescence. In the presence of single-stranded DNA (ssDNA) or the dyes are free in solution, they will not fluoresce. After completion of the amplification reaction, thermal cycler program generated a melt curve by increasing the temperature in small increments and monitoring the fluorescent signal at each step. When 50% of a dsDNA are separate into ssDNA so-called melting temperature (Tm). The difference size or GC content of PCR products demonstrated the difference Tm peak. Therefore, the multiplex GAP-PCR with melting curve analysis was developed for α-thalassemia genotyping. The primers were designed to specifically amplify two deletion fragments, the –SEA and –THAI deletions and two normal fragments, ψζ- and α2-globin gene. The melting curve analysis is able to distinguish α-thalassemia 1 heterozygotes, α-thalassemia 2 homozygotes, Hb H disease, and α-thalassemia 1 homozygote (Hb Bart’s hydrops fetalis) as shown in Figure 6 (Munkongdee et al., 2010).

The second, probe-based assays are now widely used for detection of point mutations. TaqMan assays is fluorescently labeled oligonucleotide probe. The TaqMan assay used the 5′-exonuclease activity of thermostable Taq polymerases. The probe composed a fluorescent reporter at the 5′ end and a quencher at the 3′ end. The fluorescence of the reporter is quenched due to its proximity to the quencher. However, during the annealing/extension step in the PCR reaction, the probe hybridize to the target region. The 5′ to 3′ exonuclease activity of Taq will cleave off the reporter resulting fluorescence signal which is proportional to the amount of PCR product in the sample. This technique can apply for β-thalassemia diagnosis, the multiplex probe-based fluorescence melting curve analysis (FMCA) which is a powerful tool for point mutations detection based on the Tm generated by thermal denaturation of the probe-target hybrid (Huang et al., 2011; Qiuying et al., 2011; Xiong et al., 2011).

## Direct DNA Sequencing

The mutation(s) can be identified by sequencing the PCR product, usually employing the Sanger’s dideoxy termination method. This requires the production of a single DNA strand as a template. There are a number of techniques to achieve this. An aliquot of the amplified DNA can be subjected to another round of PCR but in the presence of a single primer strand; or the original PCR product can be denatured and rapidly cooled so that the two strands remain separated; or one of the primer strand is phosphorylated at the 5′-terminus and the PCR product treated with lambda exonuclease which digests 5′-phosphorylated strand in a dsDNA; or biotin can be incorporated into the 5′-terminus of one of the primer strand enabling the PCR product to be adsorbed onto streptavidin-coated magnetic beads, which can then be treated to denature the duplexes and allow removal of the non-biotinylated strands from the beads. Alternatively, the PCR products can be subcloned into a sequencing vector, but this method introduces the possibility of detecting PCR artifacts. It enables the identification of novel or rare mutations present in the population (Sirichotiyakul et al., 2003; Korf and Rehm, 2013).

## Multiplex Ligation-Dependent Probe Amplification

Multiple ligation-dependent probe amplification (MLPA) is a multiplex PCR method that allows the detection of any deletions or duplications in the screened regions. This technique has been proven to find known and unknown deletions in unsolved cases after performing conventional techniques. The MLPA technique is easy to use and requires only a thermocycler and CE equipment (Schouten et al., 2002; Lei et al., 2019).

MLPA was started with DNA denaturation/hybridization step. DNA was denatured and incubated with a mixture of MLPA probes. MLPA probes consist of two separate oligonucleotides (LPO and RPO). The two probe oligonucleotides were hybridized to adjacent target sequences. Then, the probes can be ligated during the ligation reaction. Only ligated probes will be amplified during the PCR reaction, the number of probe ligation products is a measure for the number of target sequences in the sample. The amplification products were separated using CE.

## Next-Generation Sequencing

Advancement of sequencing technology makes an enormous contribution in characterization of the human genome. NGS technologies have gained the capacity to sequence entire human genome in an ultra-high throughput, scalability, and speed manner at a level that is not possible using Sanger sequencing technology. Most NGS platforms have three general steps: first library preparation using random fragmentation of DNA followed by ligation with custom linkers. Second, library amplification using clonal amplification methods and PCR. Third, sequencing using incorporation of fluorescent-labeled nucleotides by DNA polymerases or ligation processes. NGS has enabled researchers to diagnose and understand complex diseases through whole-genome sequencing, exome sequencing, or targeted gene panels (Yang et al., 2013; Stark et al., 2016). Recently, NGS has been applied for thalassemia screening (He et al., 2017; Shang et al., 2017; Zhang et al., 2019). Target NGS approach was designed to cover entire globin genes coding regions, their key regulatory regions, and modifier genes such as *KLF1*, *BCL11A*, *HBS1L*, and *MYB*. Preliminary data show that NGS may be much more accurate than convention thalassemia diagnosis by complete blood count (CBC), Hb analysis, Hb typing, and selected for genotyping. Preliminary study by PCR-NGS among 57,229 cases was performed in Guangxi, China, and revealed uncommon or novel mutations that could not be detected by conventional methods 458 mutations (Tables 4, 5).

**TABLE 4:** The genotype results by PCR-NGS among 57,229 cases in Baise, Guangxi, China.

Number of samples	α-Thalassemia	β-Thalassemia	α-Thalassemia with β-thalassemia	Uncomom/novel mutation (not detected by conventional methods)	Hb variants
57,229	10,018	3,141	931	458	479

**TABLE 5:** Abnormal hemoglobin detected by NGS analysis in Baise, Guangxi, China.

Hb variants	No. of Samples
Hb Hekinan II	366
Hb J-Wenchang-Wuming	28
Hb Groene Hart	15
Hb Macarena	14
Hb Owari	12
Hb Q-Thailand	11
Hb New York	8
Hb G-Coushatta	6
Hb Saclay	6
Hb Handsw	3
Hb Iraq-Halabja	3
Hb G-Honolulu	2
Hb Greenville-NC	1
Hb J-Broussais	1
Hb J-Lome	1
Hb Parma [A2]	1
Hb Prato	1
Hb Pressath	1
Hb Riccarton – II	1
Hb Wurzburg	1

## Conclusion

Laboratory diagnosis of thalassemia requires a number of tests including red blood cell indices and Hb and DNA analyses. Although low MCV and MCH are a character of thalassemic red blood cells, however, these two red blood cell indices cannot discriminate between thalassemia trait and iron deficiency or between α- and β-thalassemic conditions. Today, Hb analysis may be carried out by either HPLC or CE system. Both qualitative and quantitative analysis for Hb components can obtain from these automatic systems and help to do both prenatal and postnatal diagnoses of thalassemia within a few minutes. DNA analysis have been used for point mutation detection in β-thalassemia and large-deletion detection in α-thalassemia. Limitations of conventional thalassemia diagnosis methods are missed diagnoses due to normal or borderline red blood cell indices and/or Hb A2 levels, various labor-intensive methods may need to identify disease-causing mutation for thalassemia that have more than 1,800 mutations ranging from point mutation to large deletion. Recently, NGS has been introduced to screen for thalassemia. More loci including genetic modifiers which have significant effects on clinical manifestation should be covered in the NGS screening, which is important for precise diagnosis and treatment of thalassemia. However, there are still some limitations of NGS techniques including expensive cost. All service labs were highly recommended to select the technique(s) they are most familiar and most economic one for their daily use.

## Author Contributions

TM, KP, and PC performed the relevant literature search and wrote the review manuscript. PW and SF reviewed the manuscript for submission and provided additional points for discussion.

## Conflict of Interest

The authors declare that the research was conducted in the absence of any commercial or financial relationships that could be construed as a potential conflict of interest.

## References

[B1] Barbara J. B. (2006). “The α, β, (and (thalassemias and related conditions,” in *Haemoglobinopathy Diagnosis*, ed. Barbara J. B. (London: Blackwell), 63–138.

[B2] Borbely N. Phelan L. Szydlo R. Bain B. (2013). Capillary zone electrophoresis for haemoglobinopathy diagnosis. *J. Clin. Pathol.* 66 29–39. 10.1136/jclinpath-2012-200946 23105123

[B3] Calzolari R. McMorrow T. Yannoutsos N. Langeveld A. Grosveld F. (1999). Deletion of a region that is a candidate for the difference between the deletion forms of hereditary persistence of fetal hemoglobin and deltabeta-thalassemia affects beta- but not gamma-globin gene expression. *EMBO J.* 18 949–958. 10.1093/emboj/18.4.949 10022837 PMC1171187

[B4] Eisenstein B. I. (1990). The polymerase chain reaction. A new method for using molecular genetics for medical diagnosis. *N. Engl. J. Med.* 322 178–183. 10.1056/NEJM199001183220307 2403656

[B5] England J. M. Fraser P. M. (1973). Differentiation of iron deficiency from thalassaemia trait by routine blood-count. *Lancet* 3 449–452. 10.1016/s0140-6736(73)91878-3 4120365

[B6] Fucharoen S. Winichagoon P. (1997). Hemoglobinopathies in Southeast Asia: molecular biology and clinical medicine. *Hemoglobin* 21 299–319. 10.3109/03630269709000664 9255610

[B7] Fucharoen S. Winichagoon P. Thonglairuam V. (1998a). Beta-thalassemia associated with alpha-thalassemia in Thailand. *Hemoglobin* 12 581–592. 10.3109/03630268808991648 3209400

[B8] Fucharoen S. Winichagoon P. Wisedpanichkij R. Sae-Ngow B. Sriphanich R. Oncoung W. (1998b). Prenatal and postnatal diagnoses of thalassemias and hemoglobinopathies by HPLC. *Clin. Chem.* 44 740–748. 9554484

[B9] Galanello R. Ruggeri R. Paglietti E. Addis M. Melis M. A. Cao A. (1983). A family with segregating triplicated a globin loci and β-thalassemia. *Blood* 62 1035–1040. 6313095

[B10] HbVar, (2019). *[Internet]: A Database of Human Hemoglobin Variants and Thalassemias.* Available at: http://globin.bx.psu.edu/cgi-bin/hbvar/counter (accessed August 13, 2019).

[B11] He J. Song W. Yang J. Lu S. Yuan Y. Guo J. (2017). Next-generation sequencing improves thalassemia carrier screening among premarital adults in a high prevalence population: the Dai nationality, China. *Genet. Med.* 19 1022–1031. 10.1038/gim.2016.218 28125089

[B12] Huang Q. Liu Z. Liao Y. Chen X. Zhang Y. Li Q. (2011). Multiplex fluorescence melting curve analysis for mutation detection with dual-labeled, self-quenched probes. *PLoS One* 6:e0019206 10.1371/journal.pone.0019206 PMC3084284 21552536

[B13] Huisman T. Carver M. Baysal E. (1997). *A Syllabus of Thalassemia Mutations.* Sundsvall: The sickle cell anemia foundation.

[B14] Kanavakis E. Wainscoat J. S. Wood W. G. Weatherall D. J. Cao A. Furbetta M. (1982). The interaction of a thalassaemia with heterozygous β thalassaemia. *Br. J. Haematol.* 52 465–473. 6289863 10.1111/j.1365-2141.1982.tb03916.x

[B15] Khongthai K. Ruengdit C. Panyasai S. Pornprasert S. (2019). Analysis of deletional Hb H diseases in samples with Hb A2-Hb H and Hb A2-Hb bart’s on capillary electrophoresis. *Hemoglobin* 5 1–4. 10.1080/03630269.2019.1683573 31687860

[B16] Kingchaiyaphum B. Sanchaisuriya K. Fucharoen G. Chaibunruang A. Hess S. Y. Hinnouho G. M. (2020). Hemoglobins F, A2, and E levels in Laotian children aged 6-23 months with Hb E disorders: effect of age, sex, and thalassemia types. *Int. J. Lab. Hematol.* [Epub ahead of print] 10.1111/ijlh.13164 PMC7318314 32048804

[B17] Korf B. R. Rehm H. L. (2013). New approaches to molecular diagnosis. *JAMA* 309 1511–1521. 23571590 10.1001/jama.2013.3239

[B18] Lei Y. L. Sui H. Liu Y. J. Pan J. J. Liu Y. H. Lou J. W. (2019). Molecular and hematological characterization of a novel translation initiation codon mutation of the α2-Globin Gene (ATG>ATC or HBA2: c.3G>C). *Hemoglobin* 5 1–4. 10.1080/03630269.2019.1686012 31690131

[B19] Li Y. Liang L. Tian M. Qing T. Wu X. (2019). Electrophoresis features and genotypes of Hb bart’s hydrops fetalis. *Scand. J. Clin. Lab. Invest.* 14 1–4. 10.1080/00365513.2019.1703211 31841045

[B20] Li Z. Shang X. Luo S. Zhu F. Wei X. Zhou W. (2020). Characterization of two novel Alu element-mediated α-globin gene cluster deletions causing α0-thalassemia by targeted next-generation sequencing. *Mol. Genet. Genomics* 295 505–514. 10.1007/s00438-019-01637-w 31897801

[B21] Maggio A. Giambona A. Cai S. P. Wall J. Kan Y. W. Chehab F. F. (1993). Rapid and simultaneous typing of hemoglobin S, hemoglobin C, and seven Mediterranean β-thalassemia mutations by covalent reverse dot-blot analysis: application to prenatal diagnosis in Sicily. *Blood* 81 239–242. 8417793

[B22] Munkongdee T. Pichanun D. Butthep P. Klamchuen S. Chalermpolprapa V. Winichagoon P. (2011). Quantitative analysis of Hb Bart’s in cord blood by capillary electrophoresis system. *Ann. Hematol.* 90 741–746. 10.1007/s00277-010-1137-4 21188378

[B23] Munkongdee T. Vattanaviboon P. Thummarati P. Sewamart P. Winichagoon P. Fucharoen S. (2010). Rapid diagnosis of alpha-thalassemia by melting curve analysis. *J. Mol. Diagn.* 12 354–358. 10.2353/jmoldx.2010.090136 20190015 PMC2860472

[B24] Olivieri N. F. (1999). The beta-thalassemias. *N. Engl. J. Med.* 341 99–109. 10395635 10.1056/NEJM199907083410207

[B25] Qiuying H. Zanzan L. Yiqun L. Xiaoyun C. Yi Z. Qingge L. (2011). Multiplex fluorescence melting curve analysis for mutation detection with dual-labeled, self-quenched probes. *PLoS One* 6:e19206 10.1371/journal.pone.0019206 PMC3084284 21552536

[B26] Ruengdit C. Panyasai S. Kunyanone N. Phornsiricharoenphant W. Ngamphiw C. Tongsima S. (2020). Characterization and identification of Hb Bart’s hydrops fetalis caused by a compound heterozygous mutation –SEA /–CR, a novel α0 -thalassemia deletion. *Int. J. Lab. Hematol.* [Epub ahead of print] 10.1111/ijlh.13154 31943793

[B27] Schouten J. P. McElgunn C. J. Waaijer R. Zwijnenburg D. Diepvens F. Pals G. (2002). Relative quantification of 40 nucleic acid sequences by multiplex ligation-dependent probe amplification. *Nucleic Acids Res.* 30:e57 10.1093/nar/gnf056 PMC117299 12060695

[B28] Shang X. Peng Z. Ye Y. Asan, Zhang X. Chen Y. (2017). Rapid targeted next-generation sequencing platform for molecular screening and clinical genotyping in subjects with hemoglobinopathies. *EBioMedicine* 23 150–159. 10.1016/j.ebiom.2017.08.015 28865746 PMC5605365

[B29] Singh N. Hira J. K. Chhabra S. Khadwal A. R. Das R. Sharma P. (2020). Misdiagnosis of double heterozygous εGγ(Aγδβ)0-thalassemia/β++ thalassemia as homozygous β-thalassemia: a pitfall for molecular diagnostic laboratories. *Blood Cells Mol. Dis.* 81:102394 10.1016/j.bcmd.2019.102394 31821987

[B30] Sirichotiyakul S. Saetung R. Sanguansermsri T. (2003). Analysis of β-thalassemia mutations in northern Thailand using an automated fluorescence DNA sequencing technique. *Hemoglobin* 27 89–95. 10.1081/hem-120021541 12779270

[B31] Sripichai O. Fucharoen S. (2016). Fetal hemoglobin regulation in β-thalassemia: heterogeneity, modifiers and therapeutic approaches. *Expert Rev. Hematol.* 9 1129–1137. 10.1080/17474086.2016.1255142 27801605

[B32] Stark Z. Tan T. Y. Chong B. Brett G. R. Yap P. Walsh M. (2016). A prospective evaluation of whole-exome sequencing as a first-tier molecular test in infants with suspected monogenic disorders. *Genet. Med.* 18 1090–1096. 10.1038/gim.2016.1 26938784

[B33] Stephens A. D. Colah R. Fucharoen S. Hoyer J. Keren D. McFarlane A. (2015). ICSH recommendations for assessing automated high-performance liquid chromatography and capillary electrophoresis equipment for the quantitation of HbA2. *Int. J. Lab. Hematol.* 37 577–582. 10.1111/ijlh.12413 26372049

[B34] Suwannakhon N. Pangeson T. Seeratanachot T. Mahingsa K. Pingyod A. Bumrungpakdee W. (2019). Noninvasive prenatal screening test for compound heterozygous beta thalassemia using an amplification refractory mutation system real-time polymerase chain reaction technique. *Hematol. Rep.* 18:8124 10.4081/hr.2019.8124 PMC6761473 31579144

[B35] Tan G. B. Aw T. C. Dunstan R. A. Lee S. H. (1993). Evaluation of high performance liquid chromatography for routine estimation of haemoglobins A2 and F. *J. Clin. Pathol.* 46 852–856. 10.1136/jcp.46.9.852 7693766 PMC501524

[B36] Wainscoat J. S. Kanavakis E. Wood W. G. Letsky E. A. Huehns E. R. Marsh G. W. (1983). Thalassaemia intermedia in Cyprus: the interaction of a and β-thalassaemia. *Br. J. Haematol.* 53 411–416. 10.1111/j.1365-2141.1983.tb02041.x 6297530

[B37] Wang X. Xu J. Z. Conrey A. Mendelsohn L. Shriner D. Pirooznia M. (2020). Whole genome sequence-based haplotypes reveal a single origin of the 1393 bp HBB deletion. *J. Med. Genet.* [Epub ahead of print] 10.1136/jmedgenet-2019-106698 PMC10692763 32001505

[B38] Weatherall D. J. (1998). Pathophysiology of thalassaemia. *Baillieres Clin. Haematol.* 11 127–146. 10872475 10.1016/s0950-3536(98)80072-3

[B39] Weatherall D. J. Clegg J. B. (2001). *The Thalassaemia Syndromes*, 4th Edn Oxford, U.K: Blackwell Science.

[B40] Winichagoon P. Fucharoen S. Siritanaratkul N. (1995). Prenatal diagnosis of β-thalassemia syndromes using HRP-labeled oligonucleotide probes at Siriraj Hospital. *SE Asian J. Trop. Med. Public Health* 26(Suppl. 1), 282–286. 8629125

[B41] Winichagoon P. Fucharoen S. Weatherall D. Wasi P. (1985). Concomitant inheritance of a-thalassemia in β0-thalassemia/Hb E disease. *Am. J. Hematol.* 20 217–222. 2998183 10.1002/ajh.2830200303

[B42] Winichagoon P. Svasti S. Munkongdee T. Chaiya W. Boonmongkol P. Chantrakul N. (2008). Rapid diagnosis of thalassemias and other hemoglobinopathies by capillary electrophoresis system. *Transl. Res.* 152 178–184. 10.1016/j.trsl.2008.08.004 18940720

[B43] Xiong F. Huang Q. Chen X. Zhou Y. Zhang X. Cai R. (2011). A melting curve analysis–based PCR assay for one-step genotyping of β-thalassemia mutations a multicenter validation. *J. Mol. Diagn.* 13 427–435. 10.1016/j.jmoldx.2011.03.005 21704277 PMC3123802

[B44] Yang Y. Muzny D. M. Reid J. G. Bainbridge M. N. Willis A. Ward P. A. (2013). Clinical whole-exome sequencing for the diagnosis of mendelian disorders. *N. Engl. J. Med.* 369 1502–1511. 10.1056/NEJMoa1306555 24088041 PMC4211433

[B45] Yilmaz K. E. Acar Ö Özbas H. (2019). Missed diagnosis of β-thalassemia trait in premarital screening due to accompanying HbA2-Yialousa (HBD: c.82G>T). *J. Pediatr. Hematol. Oncol.* [Epub ahead of print] 10.1097/MPH.0000000000001633 31688628

[B46] Zhang H. Li C. Li J. Hou S. Chen D. Yan H. (2019). Next-generation sequencing improves molecular epidemiological characterization of thalassemia in Chenzhou Region, P.R. China. *J. Clin. Lab. Anal.* 33:e22845 10.1002/jcla.22845 PMC6528559 30809867

[B47] Zhao J. Li J. Lai Q. Yu Y. (2020). Combined use of gap-PCR and next-generation sequencing improves thalassaemia carrier screening among premarital adults in China. *J. Clin. Pathol.* [Epub ahead of print] 10.1136/jclinpath-2019-206339 PMC7398480 31980563

